# Association of social vulnerability index and chimeric antigen receptor T-cell therapy administration, 2018-2023

**DOI:** 10.1093/oncolo/oyaf236

**Published:** 2025-07-24

**Authors:** Alireza Boloori, Emisa Nategh, Christopher T Su

**Affiliations:** Milgard School of Business, University of Washington Tacoma, Tacoma, WA 98402, United States; McDonough School of Business, Georgetown University, Washington, DC 20057, United States; Division of Hematology and Oncology, University of Washington, Seattle, WA 98195, United States; Fred Hutchinson Cancer Center, Seattle, WA 98109, United States

**Keywords:** chimeric antigen receptor, social vulnerability, health services accessibility, cell- and tissue-based therapy

## Abstract

Chimeric antigen receptor T-cell (CAR T) therapy for patients with relapsed/refractory hematologic malignancies demands numerous visits, which may pose challenges for patients with lower socioeconomic status (SES). The Centers for Disease Control and Prevention publishes the Social Vulnerability Index (SVI), which summarizes area-level SES factors that predict how residents respond to stressors, including a new cancer ­diagnosis. We used the nationwide MarketScan commercial and Medicare insurance claims database to analyze the association between SVI and CAR T therapy completion. We performed multivariable logistic regressions (adjusting for patient-level covariates) and found that patients with hematologic malignancies residing in areas of higher SVI (lower SES) have decreased odds of CAR T therapy completion (odds ratio [OR] 0.84 for leukemia, *P *=* *.02; OR 0.72 for lymphoma, *P *<* *.001; OR 0.70 for myeloma, *P *<* *.001). Therefore, strategies to mitigate CAR T disparities may be focused on patients living in areas with higher SVI.

## Introduction

Chimeric antigen receptor T-cell (CAR T) therapy has revolutionized the treatment of relapsed/refractory hematologic malignancies. However, the administration of CAR T therapy requires multiple healthcare visits for lymphocyte collection, product infusion, and follow-up, which can introduce geographic and financial barriers for patients.^1^ Therefore, as CAR T therapy becomes more prevalent, equitable access to therapy is important. The Centers for Disease Control and Prevention publishes the Social Vulnerability Index (SVI), which captures 16 regional factors that predict how area residents respond to natural disasters and personal stressors, including a new cancer diagnosis.^2^ The SVI domains include socioeconomic status (SES; eg, employment, health insurance, and education), household characteristics (eg, English proficiency), racial and ethnic minority status, and housing type and transportation (eg, vehicle ownership), measured at the census tract level.^3^ We employed nationwide commercial and Medicare insurance claims data to determine the association of SVI with CAR T therapy administration after the first approval of commercial CAR T in October 2017. We also adjusted for the interaction of other patient-level factors, such as age, gender, insurance type, and medical comorbidity.

## Methods

We performed a retrospective data analysis using the 2018-2023 MarketScan commercial and Medicare claims data. We identified adult and pediatric patients with hematologic malignancies (ICD-10-CM C81-C95) who completed CAR T therapy, which was defined as completion of both distinct phases of CAR T therapy (collection/processing and infusion). Patients who completed lymphocyte collection but did not ultimately proceed to product infusion were not included. Determination of CAR T administration from claims was based on procedure, diagnosis, and revenue codes, guided by procedures published by the American Society for Transplantation and Cellular Therapy (**Table S1**).^4^ The aggregate SVI of the metropolitan statistical area (MSA), the lowest geographic level available in MarketScan, was used as the patient residence SVI.

We performed multiple multivariable logistic regressions, with the dependent variable being completion of CAR T therapy and the primary predictor variable being SVI (continuous from 0 to 1, higher SVI correlates to lower SES). We adjusted for covariates including age, gender, Elixhauser comorbidity index (using the van Walraven algorithm^5^), insurance type, employment status, and geographic region. Due to the varying indications of CAR T among hematologic malignancies, we stratified the analyses by leukemia, lymphoma, and multiple myeloma. All regressions were performed using SAS (version 9.4) and R (version 4.2.0).

## Results

A total of 125,751 patients with hematologic malignancies were identified, with 46,064, 60,488, and 19,199 patients diagnosed with leukemia (ICD-10-CM C91-95), lymphoma (C81-88), and myeloma (C90), respectively. Of these, 6,964 (15%), 14,093 (23%), and 5,361 (28%) of patients with leukemia, lymphoma, and myeloma completed CAR T therapy from 2018 to 2023. In multivariable logistic regression, SVI, the primary predictor of interest, was significantly associated with CAR T therapy administration. Across all disease groups, higher SVI (lower SES) is associated with decreased odd ratios (OR) of CAR T therapy (**Table 1****:** OR 0.84 [95% CI: 0.72-0.97] for leukemia, *P *=* *.02; OR 0.72 [95% CI: 0.64-0.80] for lymphoma, *P *<* *.001; OR 0.70 [95% CI: 0.58-0.84] for myeloma, *P *<* *.001).

**Table 1.** Participant characteristics and multivariable logistic regression analyses for CAR T therapy administration.

	Leukemia (*N *=* *46,064)				Lymphoma (*N = *60,488)				Myeloma (*N = *19,199)			
Patient characteristic	No CAR T (*N *=* *39,100, 85%)	**CAR T(*N *=* *6964, 15%)**	Odds ratio (OR) and 95% CI	*P* value	No CAR T (*N *=* *46,395, 77%)	CAR T (*N *=* *14,093, 23%)	Odds ratio (OR) and 95% CI	*P* value	No CAR T (*N *=* *13,838, 72%)	CAR T (*N *=* *5,361, 28%)	Odds ratio (OR) and 95% CI	*P* value
SVI, mean (standard deviation)	0.49 (SD: 0.20)	0.48 (SD: 0.20)	0.84 (CI: 0.72-0.97)	.02	0.49 (SD: 0.20)	0.48 (SD: 0.20)	0.72 (CI: 0.64-0.80)	<.001	0.51 (SD: 0.20)	0.49 (SD: 0.20)	0.70 (CI: 0.58-0.84)	<.001
Age				<.001				.05				<.001
0-39	9,540 (24%)	2,202 (32%)	1.92 (CI: 1.53-2.42)		10,788 (23%)	2,957 (21%)	0.87 (CI: 0.74-1.02)		1,005 (7%)	206 (4%)	0.44 (CI: 0.34-0.57)	
40-64	21,329 (55%)	3,727 (54%)	1.33 (CI: 1.06-1.65)		28,818 (62%)	8,928 (63%)	0.92 (CI: 0.79-1.07)		9,011 (65%)	3,958 (74%)	0.95 (CI: 0.78-1.16)	
65+ (ref)	8,231 (21%)	1,035 (15%)	—-		6,789 (15%)	2,208 (16%)	—-		3,822 (28%)	1,197 (22%)	—-	
Gender				<.001				<.001				<.001
Male (ref)	21,064 (54%)	4,067 (58%)	—-		23,279 (50%)	7,781 (55%)	—-		6,893 (50%)	2,952 (55%)	—-	
Female	18,036 (46%)	2,897 (42%)	0.87 (CI: 0.83-0.92)		23,116 (50%)	6,312 (45%)	0.84 (CI: 0.80-0.87)		6,945 (50%)	2,409 (45%)	0.84 (CI: 0.79-0.89)	
Elixhauser index/comorbidities, mean (standard deviation)	10.5 (SD: 13.2)	14.8 (SD: 12.8)	1.03 (CI: 1.03-1.04)	<.001	12.1 (SD: 12.1)	14.8 (SD: 12.4)	1.02 (CI: 1.02-1.02)	<.001	19.5 (SD: 15.4)	18.9 (SD: 14.1)	1.00 (CI: 1.00-1.00)	.51
Insurance type				<.001				.008				<.001
Commercial	30,389 (78%)	5,870 (84%)	1.82 (CI: 1.45-2.27)		39,092 (84%)	11,757 (83%)	1.23 (CI: 1.06-1.43)		9,762 (71%)	4,096 (76%)	1.46 (CI: 1.18-1.79)	
Medicare (ref)	8,711 (22%)	1,094 (16%)	—-		7,303 (16%)	2,336 (17%)	—-		4,076 (29%)	1,265 (24%)	—-	
Employment status				<.001				.48				<.001
Employed (full/part time) (ref)	22,514 (58%)	4,519 (65%)	—-		29,890 (64%)	9,041 (64%)	—-		6,691 (48%)	3,044 (57%)	—-	
Medicare-eligible retiree	4,742 (12%)	735 (11%)	1.18 (CI: 1.03-1.35)		4,571 (10%)	1,515 (11%)	0.96 (CI: 0.87-1.05)		2,230 (16%)	790 (15%)	0.99 (CI: 0.86-1.13)	
Others^a^	11,844 (30%)	1,710 (25%)	0.82 (CI: 0.77-0.88)		11,934 (26%)	3,537 (25%)	0.98 (CI: 0.93-1.02)		4,917 (36%)	1,527 (29%)	0.76 (CI: 0.71-0.82)	
Geographic region^b^				<.001				<.001				.003
South	13,824 (35%)	2,702 (39%)	1.05 (CI: 0.97-1.13)		17,253 (37%)	5,491 (39%)	1.00 (CI: 0.94-1.05)		5,289 (38%)	2,067 (39%)	0.91 (CI: 0.83-1.00)	
North Central	7,116 (18%)	1,399 (20%)	1.04 (CI: 0.94-1.14)		8,427 (18%)	2,782 (20%)	0.95 (CI: 0.89-1.02)		2,319 (17%)	985 (18%)	0.92 (CI: 0.82-1.04)	
North East	11,257 (29%)	1,584 (23%)	0.81 (CI: 0.75-0.89)		12,526 (27%)	3,247 (23%)	0.79 (CI: 0.74-0.84)		4,120 (30%)	1,382 (26%)	0.83 (CI: 0.75-0.92)	
West (ref)	6,903 (18%)	1,279 (18%)	—-		8,189 (18%)	2,573 (18%)	—-		2,110 (15%)	927 (17%)	—-	

a Includes early retiree, retiree (status unknown), COBRA continuee, long-term disability, and surviving spouse/dependent.

b South: AL, AR, DE, FL, GA, KY, LA, MD, MS, NC, OK, SC, TN, TX, VA, WV. North Central: IL, IN, IA, KS, MI, MN, MO, NE, ND, OH, SD, WI. North East: CT, ME, MA, NH, NJ, NY, PA, RI, VT. West: AK, AZ, CA, CO, HI, ID, MT, NV, NM, OR, UT, WA, WY.

Among the patient-level covariates, female gender is associated with decreased odds of CAR T therapy (OR 0.84-0.87 across all disease groups, *P *<* *.001), and commercial insurance is associated with increased odds of CAR T therapy (OR 1.23-1.82 across all disease groups, *P *<* *.001 for leukemia and myeloma, *P *=* *.008 for lymphoma). The likelihood of CAR T therapy varied by age across the disease groups, with younger patients with leukemia more likely to receive CAR T (*P *<* *.001) and older patients with lymphoma (*P = *.05) and myeloma (*P < *.001) more likely to receive CAR T. Higher comorbidity (higher Elixhauser index) is associated with increased odds of receiving CAR T for both patients with leukemia (*P *<* *.001) and lymphoma (*P *<* *.001), but not myeloma (*P *=* *.51). Finally, there is significant heterogeneity among geographic regions in which CAR T is administered (*P *<* *.001 for leukemia and lymphoma, *P *=* *.003 for myeloma).

**Figure 1.**
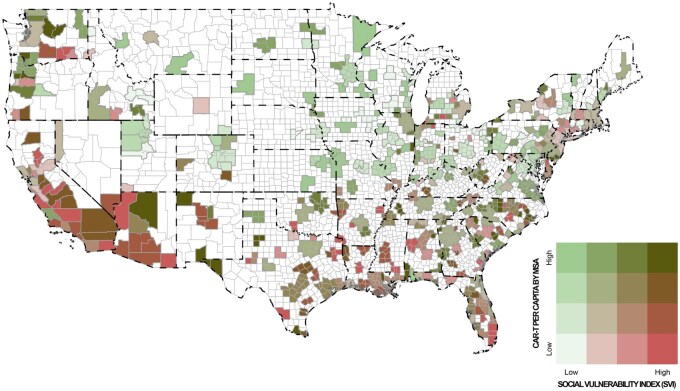
CAR T per capita and SVI, at the metropolitan statistical area level.

Additionally, we created a national plot overlaying geographic SVI with the per-capita CAR T received in each MSA, which is shown in Figure 1.

## Discussion

In our nationwide analysis of commercial and Medicare claims, patients residing in areas with lower SES (higher SVI) were less likely to receive CAR T therapy. However, Figure 1 suggests that areas surrounding urban centers generally have higher access to CAR T independent of local SVI, consistent with research on urban/rural disparities in CAR T therapy^6^ and suggesting that geographic proximity may mitigate area-level SES differences.^7^ Nonetheless, this area-level disparity in CAR T therapy completion persists after adjustment for patient-level factors, arguing for recognition of the geographic challenges in CAR T care delivery.

On the patient level, while gender disparities in CAR T access had been described previously,^6^^,^^8^ our finding that patients with commercial insurance were more likely to complete CAR T complements center-level analyses that demonstrate patients with commercial insurance have expedited access time from diagnosis to CAR T infusion.^9^ Other patient-level associations should be interpreted within the context of disease subtype, as we included a wide range of diagnosis codes for leukemia and lymphoma to avoid missing CAR T cases due to miscoding. For instance, associations of age and medical comorbidity are likely driven by differences in FDA indication for CAR T in leukemia and the diversity of leukemia and lymphoma subtypes, respectively.

While this study captures nationwide data, there were some major limitations. First, retrospective analyses of insurance claims do not contain nuanced clinical data that clinicians use in deciding whether to administer CAR T therapy. Second, MarketScan has inherent limitations, including the lack of Medicaid enrollees and information regarding race, ethnicity, and medical facilities. Finally, we were limited in assessing changes of access disparities over time, as the data only covered 6 years and 2 of the 6 CAR T therapies examined were approved for less than 3 years (after 2020).

With CAR T administration increasingly shifting to the outpatient setting and the proliferation of bispecific T-cell engager (BiTE) therapies, patients with relapsed/refractory hematologic malignancies residing in areas of access disparities have many new options for treatment. Future studies might examine differences in access between CAR T and BiTE therapy, as well as difference-in-difference analyses of cellular therapy access disparities over time. Nonetheless, our findings argue for increased recognition of area-level disparity in cellular therapy care delivery for patients with hematologic malignancies residing in rural regions and areas of lower SES.^10^

## Supplementary Material

oyaf236_Supplementary_Data

## Data Availability

The data used in this analysis is proprietary but is available from Merative.
