# Supplementary material for: Association of social vulnerability index and chimeric antigen receptor T-cell therapy administration, 2018-2023
Source: Oncologist. 2025 Jul 24;30(9):oyaf236. doi: 10.1093/oncolo/oyaf236 (PMC12422313; doi:10.1093/oncolo/oyaf236)
Supplement: oyaf236_Supplementary_Data [file oyaf236_supplementary_data.zip › CAR-T SES Supplemental Materials.docx]

**Supplemental Table 1.** Procedure, diagnosis, and revenue codes used to identify CAR T administration, extracted from the American Society for Transplantation and Cellular Therapy coding and billing guide.

| **Procedure codes** | **Revenue codes** | **Diagnosis codes** |
| --- | --- | --- |
| 'XW033C3' 'XW043C3' 'XW23346' 'XW24346' 'XW23376' 'XW24376' '0540T'  'XW033A7' 'XW043A7' 'XW033C7' 'XW043C7' 'XW033G7' 'XW043G7' 'XW033H7' 'XW043H7' 'XW033J7' 'XW043J7' 'XW033K7' 'XW043K7' 'XW033M7' 'XW043M7' 'XW033N7' 'XW043N7'  '92554' '96413' '96415' '38999' | '0874' '0335' | 'Z5112' |
